# Integrating genome-wide association studies and population genomics analysis reveals the genetic architecture of growth and backfat traits in pigs

**DOI:** 10.3389/fgene.2022.1078696

**Published:** 2022-11-25

**Authors:** Liangyu Shi, Ligang Wang, Lingzhao Fang, Mianyan Li, Jingjing Tian, Lixian Wang, Fuping Zhao

**Affiliations:** ^1^ Key Laboratory of Animal Genetics, Breeding and Reproduction (Poultry) of Ministry of Agriculture and Rural Affairs, Institute of Animal Sciences, Chinese Academy of Agricultural Sciences, Beijing, China; ^2^ Laboratory of Genetic Breeding, Reproduction and Precision Livestock Farming, School of Animal Science and Nutritional Engineering, Wuhan Polytechnic University, Wuhan, China; ^3^ Center for Quantitative Genetics and Genomics, Aarhus University, Aarhus, Denmark

**Keywords:** pigs, GWAS, growth, backfat thickness, selection signatures

## Abstract

Growth and fat deposition are complex traits, which can affect economical income in the pig industry. Due to the intensive artificial selection, a significant genetic improvement has been observed for growth and fat deposition in pigs. Here, we first investigated genomic-wide association studies (GWAS) and population genomics (e.g., selection signature) to explore the genetic basis of such complex traits in two Large White pig lines (*n* = 3,727) with the GeneSeek GGP Porcine HD array (*n* = 50,915 SNPs). Ten genetic variants were identified to be associated with growth and fatness traits in two Large White pig lines from different genetic backgrounds by performing both within-population GWAS and cross-population GWAS analyses. These ten significant loci represented eight candidate genes, *i.e., NRG4*, *BATF3*, *IRS2*, *ANO1*, *ANO9*, *RNF152*, *KCNQ5*, *and EYA2*. One of them, *ANO1* gene was simultaneously identified for both two lines in *BF100* trait. Compared to single-population GWAS, cross-population GWAS was less effective for identifying SNPs with population-specific effect*,* but more powerful for detecting SNPs with population-shared effects. We further detected genomic regions specifically selected in each of two populations, but did not observe a significant enrichment for the heritability of growth and backfat traits in such regions. In summary, the candidate genes will provide an insight into the understanding of the genetic architecture of growth-related traits and backfat thickness, and may have a potential use in the genomic breeding programs in pigs.

## 1 Introduction

In the past decades, Large White pigs had experienced intensive artificial selection for the fast growth rate and a high lean percentage (Bosi and Russo, 2004; Zhang et al., 2020). Their excellent performance has led them to dominate the global pig industry. Days of age at 100 kg live weight (AGE100) has been used as the evaluation trait for growth rate. The range of AGE100 heritability is from 0.3 to 0.5, which are moderate (Johnson and Nugent, 2003). Since a strong negative genetic correlation was between backfat thickness and carcass lean percentage (Bidanel et al., 1994), backfat thickness at 100 kg (BF100) is a good predictor for carcass lean percentage in pig breeding industry (Davoli et al., 2019). Both AGE100 and BF100 are economically important traits in Large White pigs. Therefore, a better dissection of the genetic architecture of growth and fat deposition traits will benefit for breeding and genetic improvement in pigs.

Genome-wide association study (GWAS) is a powerful tool for revealing the genetic basis of quantitative traits across the whole genome, which usually use high-density SNP genotypes in livestock and poultry population. Numerous GWASs in different pig populations have successfully identified many candidate genes associated with important production traits, such as growth, carcass (Jiang et al., 2018; Bergamaschi et al., 2020) and reproductive traits (Wang Y et al., 2017; Wang et al., 2018; Chen et al., 2022). However, low reproducibility rates (Marigorta et al., 2018) and a large number of false-positive discoveries (Colhoun et al., 2003) were common among those studies. The cross-population GWAS has emerged as an efficient strategy to prioritize GWAS results for further functional follow-ups, and Mendelian randomization studies (Panagiotou et al., 2013). This method provides the optimal power to look for the effects that are homogeneous across cohorts, meanwhile it can also shed light on between-study heterogeneity (Begum et al., 2012) and reduce false-positive findings (Evangelou et al., 2007).

In this study, the objectives were 1) to conduct GWAS for AGE100 and BF100 within two Large White pig lines with distinct genetic backgrounds; 2) to detect shared loci in cross-population GWAS; 3) to integrate GWAS with selection signatures to explore whether the associated loci are under selection. The findings here will help unravel the genetic background of these two complex traits in pigs.

## 2 Materials and methods

### 2.1 Animals and phenotypes

Data were obtained from two Large White populations with different genetic backgrounds in one Chinese commercial pig company in Shanghai City, which were originated from Canadian and French lines. Feeding and performance testing of animals from these two lines were carried out at two different farms. When the average live weight per batch was approximate 100 kg, individual tests were performed. Body weight and ultrasonic backfat thickness between 11^th^ to 12^th^ ribs were measured. The initial and ending dates were recorded. To uniform the data, the measured age was adjusted to 100 kg live weight using the equation (Wang, 2007, 18–19): $AGE100=measured\;age+\frac{100\;kg-measured\;age}{CF}$ , where $CF_{male}=\frac{measured\;weight}{measured\;age}\times 1.826$ and $CF_{female}=\frac{measured\;weight}{measured\;age}\times 1.715$ , and the measured backfat thickness was adjusted to 100 kg live weight using the formula (Chen et al., 2019): $BF100_{male}=measured\;backfat\times \frac{12.402}{12.402+0.106\times \left(measured\;weight-100\right)}$ and $BF100_{female}=measured\;backfat\times \frac{13.706}{13.706+0.119\times \left(measured\;weight-100\right)}$ .

Table 1 shows a summary of the descriptive statistics of AGE100 and BF100 traits. Totally, there were 3,727 observations available for both AGE100 and BF100. Out of them, 2,138 were from the Canadian lines and 1,589 were from French lines. These Large White pigs were born between 2015 and 2020. According to the pedigree information, there were no genetic connectedness between two populations. As seen in Table1, the heritability of AGE100 in Canadian and France lines are 0.15 and 0.30, while the heritability of BF100 in Canadian and France lines are 0.21 and 0.44, respectively.

**TABLE 1:** Descriptive statistics of AGE100 and BF100.

Trait	Source	Unit	N	min	Max	Mean	Sd	*h* ^2^	Significant level
AGE100	Canadian line	day	2,138	138.25	215.78	173.04	10.83	0.15	*p* <0.001
	France line		1,589	132.89	215.75	162.66	10.77	0.30	
BF100	Canadian line	mm	2,138	5.89	21.15	11.11	2.00	0.21	*p* <0.001
	France line		1,589	5.36	19.82	10.20	2.06	0.44	

### 2.2 Genotyping and quality control

Genotyping all the individuals with phenotypes was carried out using GeneSeek GGP Porcine HD array. Since the SNP chip is composed of 50,915 probes according to the *Sus Scrofa* 10.2 version, the autosomal SNPs were further liftovered to the latest version of the pig genome *Sus Scrofa* 11.1. Thus, 46,258 autosomal SNPs were kept for the subsequent analysis.

Quality control was executed by PLINK (v1.90) (Purcell et al., 2007). Pigs with call rate < 0.9 were excluded. SNPs with a minor allele frequency (MAF) below 0.05 and call rate < 0.9 were excluded in each population. Finally, the remaining autosomal SNPs were 41,172 and 40,506 with the average distances of 53.87 Kb and 54.85 Kb between adjacent SNPs in Canadian and French lines, respectively.

### 2.3 Population genomics analysis

To investigate the population stratification, principal component analysis (PCA) was conducted using the remaining SNPs to obtain eigenvalues and eigenvectors by PLINK (v1.90) (Purcell et al., 2007). In addition, we performed ancestry estimation using ADMIXTURE (v1.3.0) (Alexander et al., 2009). The number of level of genetic structure were estimated from K = 1 to four for all individuals jointly, followed by the cross-validation error (CV) procedure. The linkage disequilibrium (LD, expressed as *r* ^*2*^) was calculated using PLINK (v1.90) (Purcell et al., 2007) within each line. In this study, the population effect size (*Ne*) was computed by *SNeP* software (v1.1) (Barbato et al., 2015).

### 2.4 Genome wide association studies

The mixed model was executed for analyzing the traits under study as following:

y=μ+Xb+Wg+Zu+e (1)

where *y* is the vector of target phenotypes of individuals; *μ* is overall mean; *b* is the vector of fixed effects: sex (two levels) and year-season in which seasons were comprised of four levels (Spring:  March to May; Summer:  June to August; Autumn: September to November; Winter:  December to February); *g* is the vector of the SNP effects, *X* is the matrix of incidence associating each observations to the pertinent level of fixed effects, *W* is the incidence matrix relating observations to SNPs effects with elements coded as 0, one and two for genotype A_1_A_1_, A_1_A_2_, and A_2_A_2_, respectively, *u* is the random additive genetic effect of the individual and is assumed to be distributed as $N\left(0,G\sigma_{u}^{2}\right)$ , G is the genomic relationship matrix and $\sigma_{u}^{2}$ is the polygenic additive genetic variance, *Z* is incidence matrix for *u*, *e* is the random residual and is assumed to be distributed as $N\left(0,I\sigma_{e}^{2}\right)$ , where *I* is the identity matrix and $\sigma_{e}^{2}$ is the residual variance. Associations between the target traits and the SNPs were analyzed using single-SNP association tests in each population, which were implemented by the *mlma* option of the software GCTA (Version 1.93.3beta) (Yang et al., 2011).

Furthermore, we integrated two Large White pig lines to identify the candidate genes using combined-population GWAS and cross-population GWAS. In the combined-population GWAS, line effect was taken into account the fixed effect, and the analysis procedure was followed the single-population GWAS mentioned above. The cross-population GWAS also utilized the summary data of single-population GWAS to implement meta-analysis by METAL (version 2011–03–25) (Willer et al., 2010). In meta-analysis, the weighted Z-score model took account of the *p*-values, direction of SNP effects and the number of individuals. In each case, threshold *p*-values were set to -log_10_ (1/SNPs) and -log_10_ (0.05/SNPs) for suggestive and Bonferroni-adjusted genome-wide significance, respectively. Quantile-quantile (QQ) plot of–log (*p*-values) was examined to determine how well GCTA accounted for population structure and family relatedness.

### 2.5 Partitioning heritabilities of complex traits based on selection signatures

In response to intensive artificial selection pressures, the porcine genome has been sculpted signals at the underlying genomic regions harboring functional genetic variants, which are termed as selection signatures (Bertolini et al., 2018). Population differentiation-based methods were performed, including F_ST_, hapFLK and runs of homozygosity (ROH). The VCFtools software (Danecek et al., 2011) was used to compute the Weir and Cockerham’s F_ST_ estimator (Weir and Cockerham, 1984) per site between the two pig lines. The hapFLK statistic (Fariello et al., 2013) was estimated using hapFLK module in python. It should be mentioned that before calculation of hapFLK values, fastPHASE (Scheet and Matthew, 2006) was used to determine the optimum number of haplotype clusters. ROH analysis was performed using PLINK (v1.90) (Purcell et al., 2007), and the parameters were assigned following our previous study (Shi et al., 2020).

In addition, we further investigated the impact of SNPs undergoing selection signatures on the traits under study. First, according to the sizes of selection signals, we sorted the whole SNPs and split them into five sets [0–20%, 20–40%, 40–60%, 60–80% and 80–100%]. To quantify the relative importance of SNPs sets, we calculated one of these SNP sets and the remaining four SNP sets explaining the proportion of phenotypic variance. The statistical model was employed as below:

$$y=\mu +Xb+u_{1}+u_{2}+e$$ (2)

where *u*_1_ is the vector of the first random additive genetic effect, which is distributed as $u_{1}∼N\left(0,G_{1}\sigma_{u_{1}}^{2}\right)$ , where $G_{1}$ is the first genomic relationship matrix that was constructed using one of quantile SNPs, *u*_2_ is the vector of second random additive genetic effect, which is distributed as $u_{2}∼N\left(0,G_{2}\sigma_{u_{2}}^{2}\right)$ , where $G_{2}$ is the second genomic relationship matrix which was calculated using the remaining four SNP sets. Other notations are same as Eq. 1. These two random effect variances can be computed by Qgg R package (Rohde et al., 2020). The proportion of phenotypic variance contributed by the selected SNP set ( $h_{u_{1}}^{2}$ ) was computed using the following equation: $h_{u_{1}}^{2}=\frac{\sigma_{u_{1}}^{2}}{\sigma_{u_{1}}^{2}+\sigma_{u_{2}}^{2}+\sigma_{e}^{2}}$ .

### 2.6 Annotation of candidate genes

To identify positional candidate genes, the BioMart database (http://www.ensembl.org/) was implemented. The candidate genes resided within the genomic regions of up- and downstream 500 kb around the significant SNPs were taken into account in our study. Functional annotation of the genes located in the regions of interest was performed with R package WebGestaltR (Wang J et al., 2017).

## 3 Results

### 3.1 Descriptive statistics of phenotype data and population structure analysis

**FIGURE 1:**
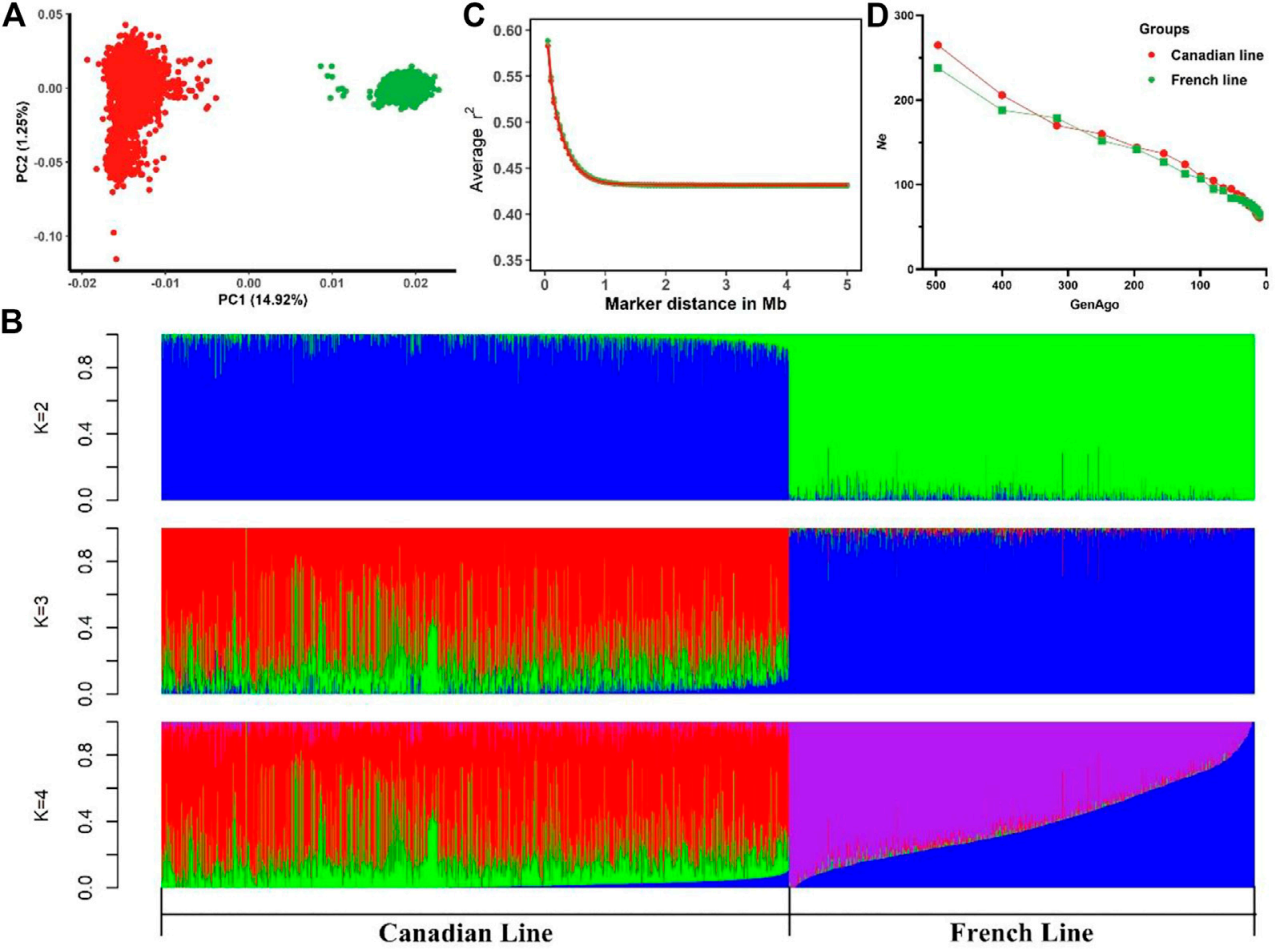
Descriptive statistics of population structure. **(A)** Principal component analysis indicating the relationship between the first two principal components (PC1, PC2) and the proportions of genetic variances explained (% explained var.) among Canadian and French pig lines. **(B)** Admixture analysis for Canadian and French lines ranging from K = 2 to K = 4. **(C)** Linkage disequilibrium (LD) for the Canadian and French lines, *r* ^2^ values were averaged within bins of 0.5 Mb between pair-wise SNP physical distance and pooled over autosomes. **(D)** Average estimated effective population size (*Ne*) plotted against the number of past 500 generations.

Table 1 summarized the descriptive statistics of AGE100 and BF100 in the two Large White pig lines. Phenotypes of both traits followed a normal distribution (Supplementary Figure S1). PCA showed a substantial genetic diversity between these two populations. The total genetic variance in these animals was explained 14.92%, 1.25% and 1.16% by the first three principal components, respectively (Figure 1A). As seen in Figure 1A, PC1 distinctly divided them into Canadian and French line, suggesting that two pig populations shared less similarity in the genetic background. Moreover, in PC2 Canadian line had a widespread. This finding agreed with the results of model-based analysis of population admixture which was showed in Figure 1B (K = 2, 3 and 4). Figure 1C displayed average LD (*r* ^*2*^) at various physical distances between two loci on all the autosomes. The average *r* ^*2*^ at pair-wise SNP distance of less than 5 Mb on autosomes ranged from 0.432 to 0.582 in Canadian Large White population. The LD decay pattern in French population was similar to the Canadian line with average *r* ^*2*^ ranging from 0.431 to 0.588. The average *r* ^*2*^ decreased much more slowly with the increase of pair-wise SNP physical distance and remained constant beyond 1 Mb in two lines (Figure 1C). *Ne* estimated at 99 generations ago were 110 for Canadian line and 107 for French line, respectively (Figure 1D). It should be noted that MAFs in Canadian and French lines had no significant differences (Supplementary Figure S2).

### 3.2 Partitioning heritability with selection signatures partitioning heritability with selection signatures

To identify the selection signatures between these two populations, F_ST_, hapFLK and ROH were used to detect selection signatures across the whole genome in two pig lines (Supplementary Figure S3). In each selection signature method, the top 20% of genomic regions were selected. All the genes in the genomic regions under selection were further analyzed for functional annotation using the WebGestaltR package (Wang J et al., 2017) (Supplementary Figure S4). To find the proportions of phenotypic variances explained by SNPs subjected to selection, the entire genome was divided into five groups according to selection signatures. The heritability of AGE100 and BF100 explained by these groups were jointly estimated using a multi-components (a GMR for each group) linear mixed model. However, we did not observe heritability of AGE100 and BF100 tended to enrich in the regions under selection (Supplementary Figure S5). The possible reason for this result was the lack of statistical power due to the small population size and the traits are complex (Ma et al., 2019).

### 3.3 SNPs significantly associated with AGE100

**FIGURE 2:**
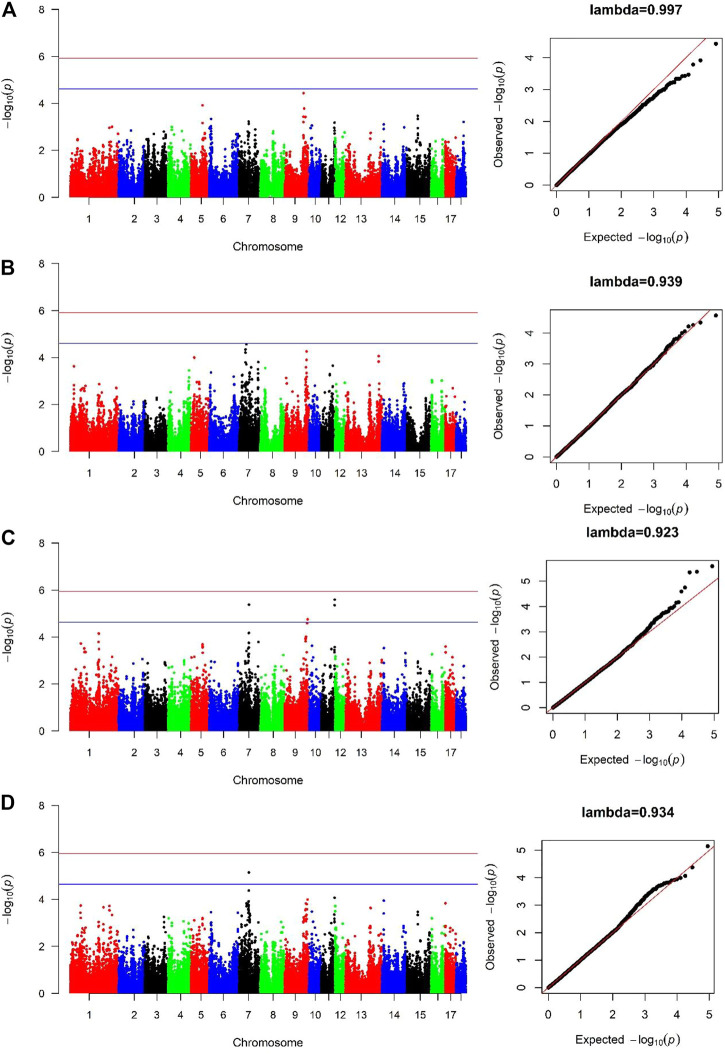
Manhattan and Quantile-Quantile (QQ) plots of genome-wide association analysis for AGE100 trait. **(A)** is Canadian line. **(B)** is French line. **(C)** is combined-population. **(D)** is cross-population GWAS analysis. The x-axis denotes autosomes. The y-axis indicates -log_10_ (*p*-values).

For both lines, no genome-wide significant SNPs were detected associated with AGE100 trait (Figure 2A, Figure 2B), while only four SNPs at the suggestive significant level were observed (Figure 2C; Table 2). Only one SNP located in *Sus scrofa* chromosome 7 (SSC7) was identified by cross-population GWAS to be significantly associated with AGE100 at the suggestive significant level, which was also detected in the combined-population (Figure 2D; Table 2).

**TABLE 2:** Significant SNPs and genes in which they are located identified in the genome-wide association study for AGE100 trait.

SSC (*Sus scrofa* chromosome)	Position (bp)	*p*-value	Distance	Gene
Combined-population				
7	56252185	4.19 × 10^–6^	Within	*NRG4*
9	130681106	1.77 × 10^–5^	Downstream 18 Kb	*BATF3*
11	76296871	4.45 × 10^–6^		
11	76606088	2.56 × 10^–6^	Downstream 20 Kb	*IRS2*
Cross-population				
7	56252185	7.13 × 10^–6^	Within	*NRG4*

### 3.4 SNPs significantly associated with BF100

**FIGURE 3:**
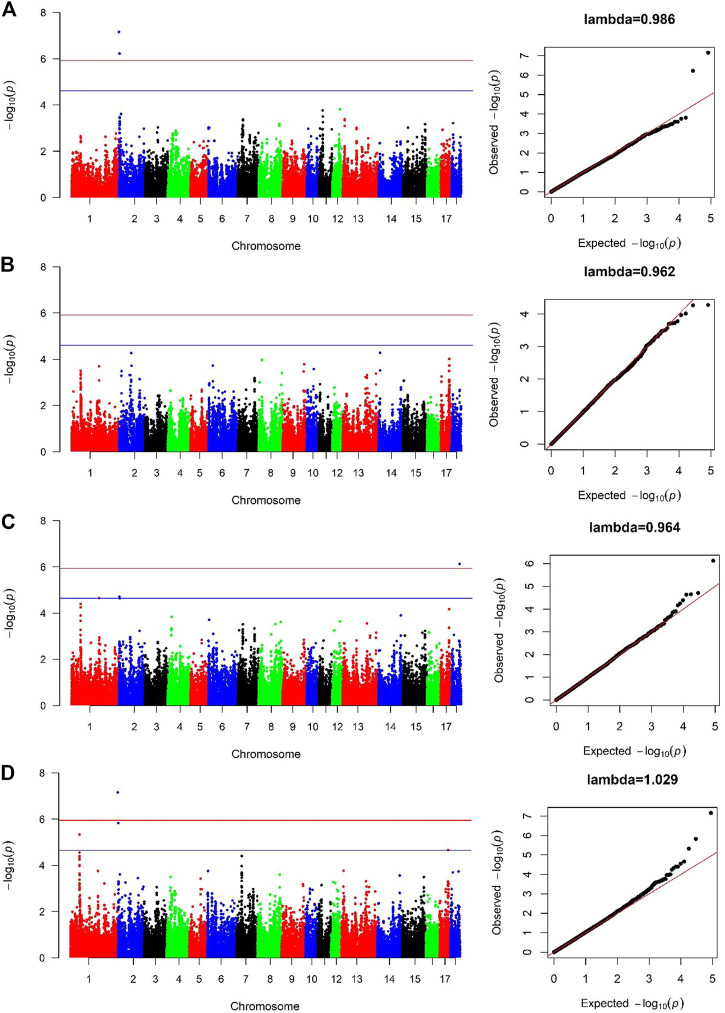
Manhattan and Quantile-Quantile (QQ) plots of genome-wide association analysis for BF100 trait. **(A)** is Canadian line. **(B)** is French line. **(C)** is combined-population. **(D)** is cross-population GWAS. The x-axis denotes autosomes. The y-axis indicates -log_10_ (*p*-values).

For Canadian line, two SNPs (SSC2:236157, SSC2:3285386) were significantly associated with BF100 at genome-wide significant level. These two SNPs were also detected by the cross-population GWAS analysis, which resided in the genic regions of *ANO9* and *ANO1* (Figure 3A; Table 3). There was no significant SNPs identified to be associated with BF100 trait in the French line (Figure 3B).

**TABLE 3:** Significant SNPs and genes in which they are located identified in the genome-wide association study for BF100 trait.

SSC	Position (bp)	*p*-value	Distance	Gene
Canadian line				
2	236157	6.99 × 10^–8^	Within	*ANO9*
2	3285386	5.98 × 10^–7^	Within	*ANO1*
Combined-population				
1	159697691	2.25 × 10^–5^	Upstream 96 Kb	*RNF152*
2	3285386	1.95 × 10^–5^	Within	*ANO1*
18	48235741	7.50 × 10^–7^		
Cross-population				
1	52528119	4.69 × 10^–6^	Within	*KCNQ5*
2	236157	6.99 × 10^–8^	Within	*ANO9*
2	3285386	1.49 × 10^–6^	Within	*ANO1*
17	49046378	2.19 × 10^–5^	Within	*EYA2*

Three SNPs (SSC1:159697691, SSC2:3285386 and SSC18:48235741) were identified to be significantly associated with BF100 using the combined-population GWAS at the suggestive significant level (Figure 3C; Table 3). Cross-population GWAS analysis of association results from the two pig populations revealed two additional SNPs for BF100, and the most significantly associated SNP from the cross-population GWAS was located on chromosome 2 (Figure 3D; Table 3).

## 4 Discussion

In present study, we carried out the population structure and admixture analyses in two different genetic background lines of Large White pigs which are Canadian and French lines. LD pattern of the studied populations strongly relied on their evolutionary history and structure (Bohmanova et al., 2010). The LD at long distances revealed *Ne* in the recent past, while the LD at short distances reflected the *Ne* in the distant past (Hayes et al., 2003). Although the LD pattern and *Ne* results in these two lines were similar, the Canadian and French lines were obviously separated by admixture analysis at assumed K values from 2 to 4, which was further supported by PCA. Therefore, these two populations could not be pooled directly to estimate SNP effects because of the population stratification. In multi-population association studies, population stratification is the confounding factor that inflates the false positive rate (Jiang et al., 2019; Sohail et al., 2019; Morris et al., 2020). The cross-population GWAS analysis was used summary statistics from single-population GWAS, which can alleviate the problem of population stratification (Salanti et al., 2005; Wang et al., 2012).

Lots of the significant SNPs detected using the single-population GWAS were validated by the cross-population GWAS. Furthermore, cross-population GWAS identified novel genetic loci. This method can produce a precise estimate of the SNP effect and increase considerably statistical power. This property is important to a small sample size because the power of the primary study is limited. Some significant SNPs were identified using the combined-population GWAS but not detected using the cross-population GWAS, although the data size is same. The reason might be that a SNP is identified to be significantly associated with a trait in one population since the SNP is in linkage with the causal mutation. Nevertheless, the LD pattern might be different in another population, and thus can lead to a weakened association between the SNP and trait if combining these populations. Moreover, different populations may have different causal variants segregating at the same locus, which can result in the reduction of significance using the cross-population GWAS, although this reversal of effect of causal variants is not common (van den Berg et al., 2020).

For AGE100, both combined-population GWAS and cross-population GWAS simultaneously identified one SNP (SSC7:56252185) that resided in the genic region of the *NRG4* gene. This gene is a member of the EGF family of extracellular ligands, and played a key role in the modulation of glucose and lipid metabolism and energy balance (Tutunchi et al., 2020; Martinez et al., 2022). In addition, four SNPs significantly associated with BF100 were identified by cross-population GWAS. Out of them, three SNPs (SSC1:52528199, SSC2:236157, and SSC2:3285386) located the genomic regions identified to be associated with backfat thickness in multiple pig lines by other study (Gozalo-Marcilla et al., 2021). In this region, both combined-population and cross-population GWAS simultaneously identified anoctamin 1 (ANO1) gene, which was also known as *TMEM16A*. *ANO*1 and *IRS*2 played positive roles in insulin secretion (Dong et al., 2006; Xu et al., 2014; Hashimoto et al., 2015; Toyoshima et al., 2020), which could affect growth rate and fat deposition in pigs. *ANO1* gene defects or its expression in pancreatic islets might influence cytokine expression and elicit an immune response that could result in death of *β* cell (Xu et al., 2014). CRISPR-edited animals study demonstrated *KCNQ3* expression was sensitive to the energy state of animals (Stincic et al., 2021). *EYA2* had been documented to be closely associated with the biological processes of striated muscle tissue development, muscle cell and skeletal muscle cell differentiation (Li et al., 2019). In addition, *RNF152* gene was also identified to be related to IMF (Zhang et al., 2021), and *EYA2* has already been identified by selection signature detection between the Sudanese thin-tail vs. Ethiopian fat-rump sheep (Ahbara et al., 2019).

## 5 Conclusion

In this study, we performed single-, combined- and cross-population GWAS analysis for growth and fatness traits in purebred Large White pigs that were from two separated foundation lines (Canadian and French lines). We demonstrated that the cross-population GWAS could be used to increase the power of GWAS. One candidate gene, *ANO1* gene was simultaneously identified for both two lines in BF100 trait. By integrating selection signatures with growth rate and backfat thickness relevant trait association studies, however, we did not observer that heritability of growth and fatness were significantly enriched in genomic regions under selection. Future study is needed to refine the genomic regions and identify candidate genes and candidate mutations affecting growth and fatness in pigs.

## Data Availability

The datasets presented in this study can be found in online repositories. Phenotype and genotype data have been uploaded on figshare website (https://doi.org/10.6084/m9.figshare.21516159).
