# Supplementary material for: Integrating genome-wide association studies and population genomics analysis reveals the genetic architecture of growth and backfat traits in pigs
Source: Front Genet. 2022 Nov 25;13:1078696. doi: 10.3389/fgene.2022.1078696 (PMC9732542; doi:10.3389/fgene.2022.1078696)
Supplement: Supplementary file 1 [file Image1.pdf]

Supplementary Material

1 Supplementary Figures

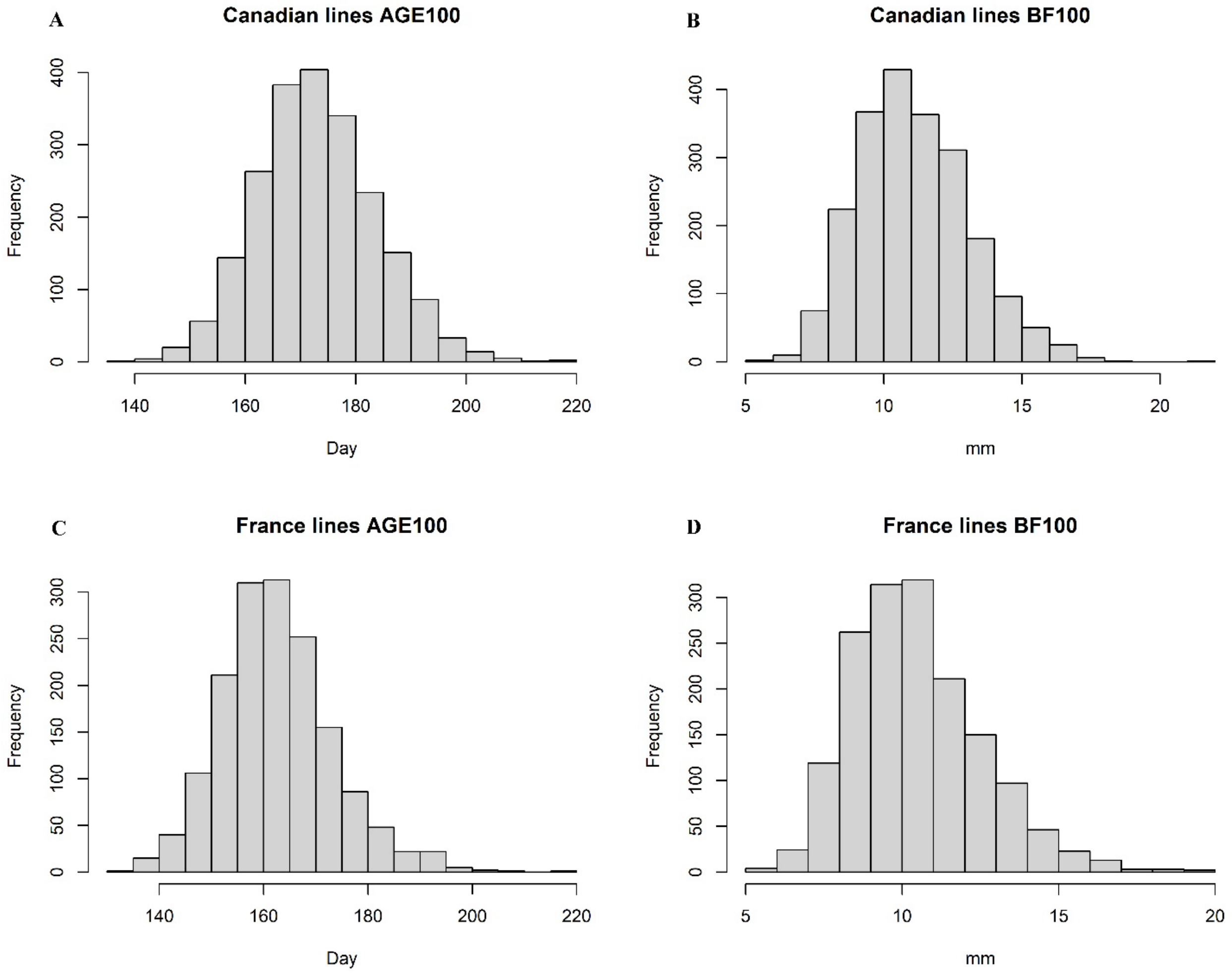

Supplementary Figure 1. The distribution of AGE100 and BF100 in the two Large White pig lines.

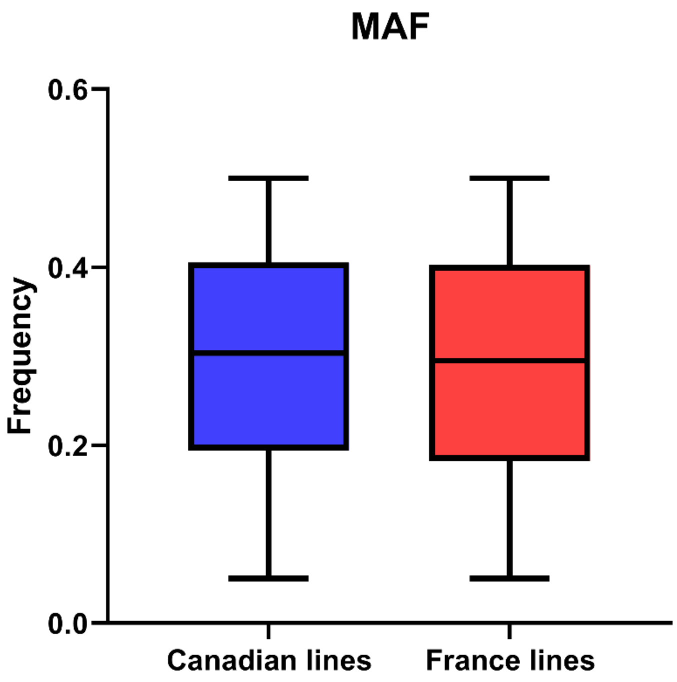

Supplementary Figure 2. MAF for Canadian and French lines.

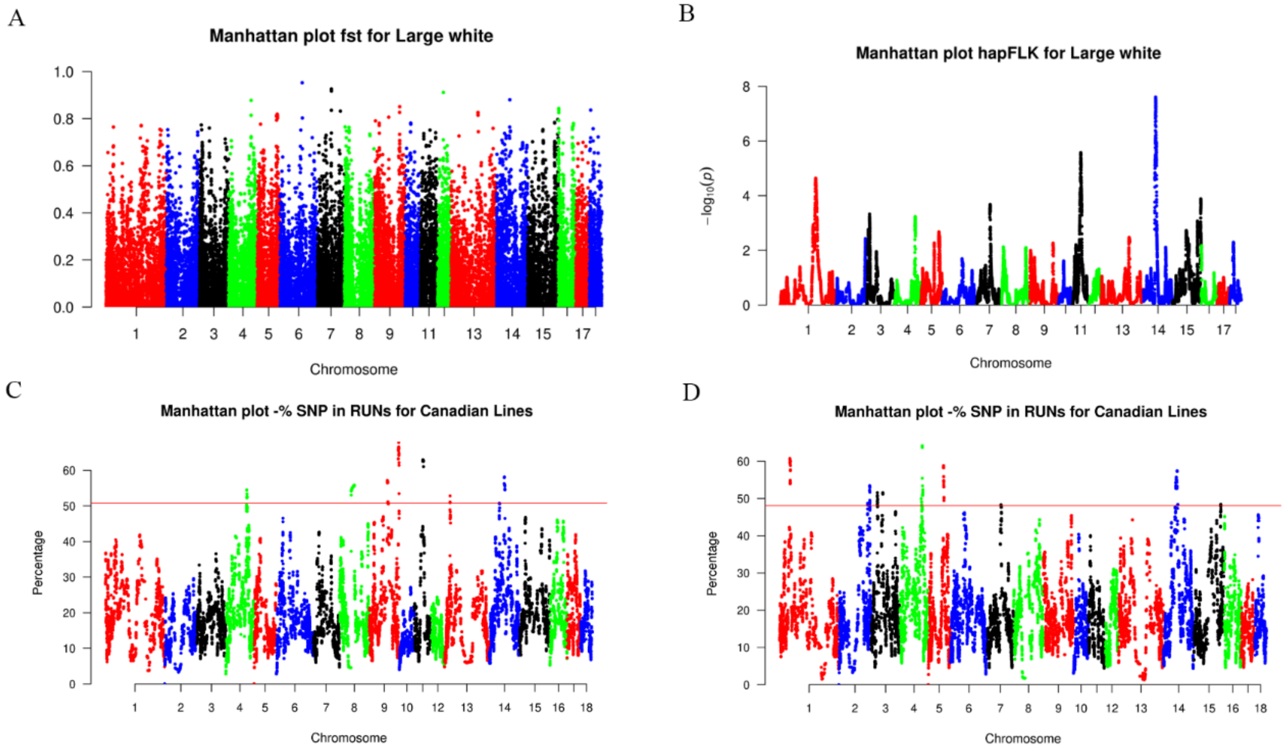

Supplementary Figure 3. Manhattan plot for selection signatures across individuals. (A) FST; (B) hapFLK; (C) ROH for Canadian lines; (D) ROH for France lines.

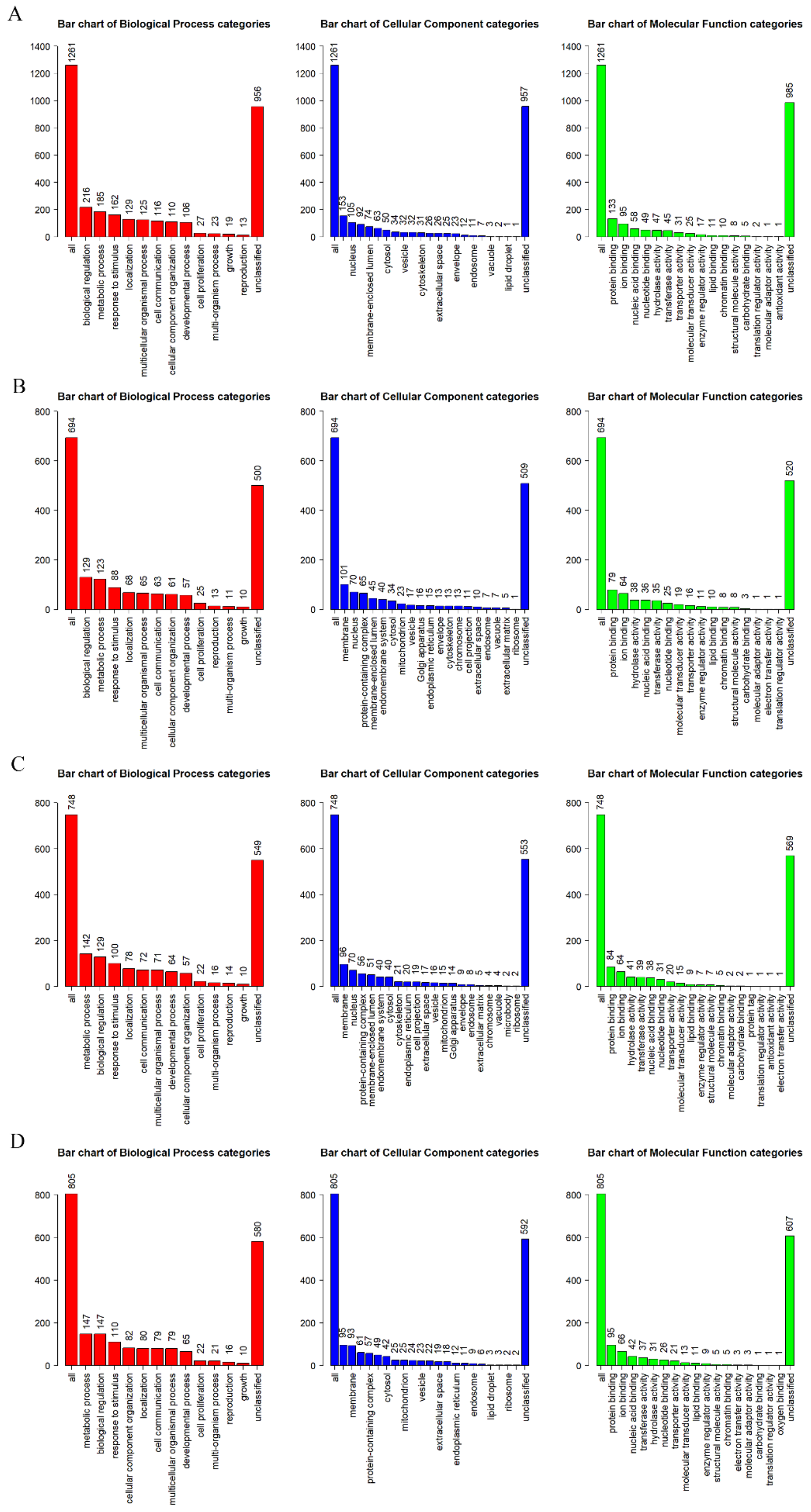

Supplementary Figure 4. GO enrichment analysis for selection signatures. (A) FST; (B) hapFLK; (C) ROH for Canadian lines; (D) ROH for France lines.

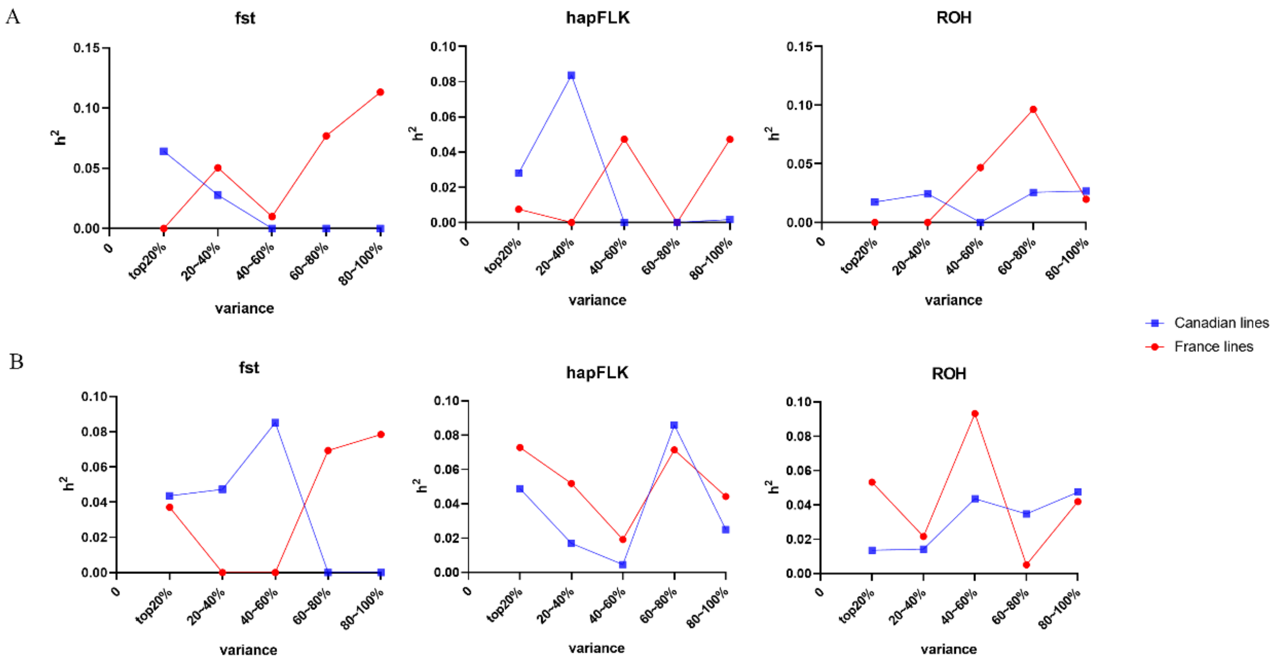

Supplementary Figure 5. Heritability with trait-specific selection signatures. (A) AGE100 trait; (B) BF100 trait.
